# Withdrawal of pharmacological treatment for heart failure in patients with recovered dilated cardiomyopathy (TRED-HF): an open-label, pilot, randomised trial

**DOI:** 10.1016/S0140-6736(18)32484-X

**Published:** 2019-01-05

**Authors:** Brian P Halliday, Rebecca Wassall, Amrit S Lota, Zohya Khalique, John Gregson, Simon Newsome, Robert Jackson, Tsveta Rahneva, Rick Wage, Gillian Smith, Lucia Venneri, Upasana Tayal, Dominique Auger, William Midwinter, Nicola Whiffin, Ronak Rajani, Jason N Dungu, Antonis Pantazis, Stuart A Cook, James S Ware, A John Baksi, Dudley J Pennell, Stuart D Rosen, Martin R Cowie, John G F Cleland, Sanjay K Prasad

**Affiliations:** a Cardiovascular Research Centre and Cardiovascular Magnetic Resonance Unit, Royal Brompton Hospital, London, UK; b National Heart and Lung Institute, Imperial College London, London, UK; c MRC London Institute of Medical Sciences, Imperial College London, London, UK; d London School of Hygiene & Tropical Medicine, London, UK; e Guy's and St Thomas' NHS Foundation Trust and King's College London, London, UK; f Basildon and Thurrock Hospitals NHS Foundation Trust, Essex, UK; g National Heart Centre Singapore, Singapore; h Department of Cardiology, Ealing Hospital, London, UK; i Robertson Centre for Biostatistics, University of Glasgow, Glasgow, UK

## Abstract

**Background:**

Patients with dilated cardiomyopathy whose symptoms and cardiac function have recovered often ask whether their medications can be stopped. The safety of withdrawing treatment in this situation is unknown.

**Methods:**

We did an open-label, pilot, randomised trial to examine the effect of phased withdrawal of heart failure medications in patients with previous dilated cardiomyopathy who were now asymptomatic, whose left ventricular ejection fraction (LVEF) had improved from less than 40% to 50% or greater, whose left ventricular end-diastolic volume (LVEDV) had normalised, and who had an N-terminal pro-B-type natriuretic peptide (NT-pro-BNP) concentration less than 250 ng/L. Patients were recruited from a network of hospitals in the UK, assessed at one centre (Royal Brompton and Harefield NHS Foundation Trust, London, UK), and randomly assigned (1:1) to phased withdrawal or continuation of treatment. After 6 months, patients in the continued treatment group had treatment withdrawn by the same method. The primary endpoint was a relapse of dilated cardiomyopathy within 6 months, defined by a reduction in LVEF of more than 10% and to less than 50%, an increase in LVEDV by more than 10% and to higher than the normal range, a two-fold rise in NT-pro-BNP concentration and to more than 400 ng/L, or clinical evidence of heart failure, at which point treatments were re-established. The primary analysis was by intention to treat. This trial is registered with ClinicalTrials.gov, number NCT02859311.

**Findings:**

Between April 21, 2016, and Aug 22, 2017, 51 patients were enrolled. 25 were randomly assigned to the treatment withdrawal group and 26 to continue treatment. Over the first 6 months, 11 (44%) patients randomly assigned to treatment withdrawal met the primary endpoint of relapse compared with none of those assigned to continue treatment (Kaplan-Meier estimate of event rate 45·7% [95% CI 28·5–67·2]; p=0·0001). After 6 months, 25 (96%) of 26 patients assigned initially to continue treatment attempted its withdrawal. During the following 6 months, nine patients met the primary endpoint of relapse (Kaplan-Meier estimate of event rate 36·0% [95% CI 20·6–57·8]). No deaths were reported in either group and three serious adverse events were reported in the treatment withdrawal group: hospital admissions for non-cardiac chest pain, sepsis, and an elective procedure.

**Interpretation:**

Many patients deemed to have recovered from dilated cardiomyopathy will relapse following treatment withdrawal. Until robust predictors of relapse are defined, treatment should continue indefinitely.

**Funding:**

British Heart Foundation, Alexander Jansons Foundation, Royal Brompton Hospital and Imperial College London, Imperial College Biomedical Research Centre, Wellcome Trust, and Rosetrees Trust.

## Introduction

Outcomes for patients with dilated cardiomyopathy vary, but for many the disease runs a benign course.^1^ An improvement in left ventricular ejection fraction (LVEF) and reduction in left ventricle size is seen in around 40% of patients.^2^ Plasma concentrations of natriuretic peptides might also normalise with treatment.^3^ Such patients are typically young with few comorbidities and have a good prognosis.^3^, ^4^, ^5^, ^6^ Although some patients with improved function remain symptomatic,^3^ those with the greatest improvement are often rendered asymptomatic.^7^

Following resolution of symptoms and recovery in cardiac function, many patients ask whether it is necessary to continue lifelong treatment, especially if they are having side-effects. Patients are often young and reluctant to take medications for many years without evidence of continued benefit. Young women are often eager to stop treatment before attempting to become pregnant. Medications also represent a substantial financial burden for patients in some countries. Reducing the number of unnecessary medications might also improve overall wellbeing of patients.

Whether patients with a previous diagnosis of dilated cardiomyopathy and clinical, imaging, and biochemical markers of recovered cardiac function benefit from continuing treatment indefinitely is unknown. Some patients with these features might have achieved permanent recovery and so continued treatment might be unnecessary. For others, relapse could occur if treatment is withdrawn. There is an absense of prospective data investigating treatment withdrawal in patients with recovered dilated cardiomyopathy and consequently no consensus among experts or clear recommendations in guidelines.^8^ Accordingly, we aimed to do a pilot study to examine the effect of treatment withdrawal in patients with clinical, imaging, and biochemical evidence of recovery from dilated cardiomyopathy.

**Research in context**

**Evidence before this study**

We searched PubMed using the terms “recovered ejection fraction”, “left ventricular reverse remodelling”, “dilated cardiomyopathy”, “therapy withdrawal”, and “treatment withdrawal” for randomised or observational studies published up to Sept 3, 2018. We did not limit the search to English language publications. Previous reports about treatment withdrawal in patients with recovered dilated cardiomyopathy have been based on retrospective case-note reviews of populations with varying levels of recovery. The need for continued pharmacological heart failure treatment in patients with recovered dilated cardiomyopathy is unclear. Patients frequently ask to stop medications and are given conflicting medical guidance.

**Added value of this study**

The results of the TRED-HF trial suggest that around four in ten patients with recovered dilated cardiomyopathy will have a relapse within 6 months of starting phased withdrawal of pharmacological treatment for heart failure.

**Implications of all the available evidence**

Data from this randomised trial suggest that treatment should not usually be withdrawn in patients with recovered dilated cardiomyopathy. If a patient wishes to initiate treatment withdrawal, cardiac function should be monitored carefully and, until more information is available, indefinitely. Further research might identify subgroups of patients in whom pharmacological treatment for heart failure can be safely withdrawn.

## Methods

### Study design and patients

We did an open-label, pilot, randomised trial of phased withdrawal of pharmacological treatment for heart failure. Investigations were done at a single centre (Royal Brompton and Harefield NHS Foundation Trust, London, UK). Patients were identified at the trial centre and participant identification centres in the UK (Guy's and St Thomas' NHS Foundation Trust, King's College Hospital NHS Foundation Trust, St George's NHS Foundation Trust, London Northwest NHS Healthcare Trust, Epsom and St Helier University Hospitals, and Basildon and Thurrock University Hospitals). All patients provided written informed consent. The possible risk of heart failure and major arrhythmia was discussed. The trial was approved by the National Research Ethics Committee (16/LO/0065), to whom annual progress reports were submitted, and given NHS Permission by the Royal Brompton and Harefield NHS Trust after review of study documentation and discussion of the risks and benefits. The trial was authorised by the Medicines and Healthcare Products Regulatory Agency and annual development safety update reports were provided. The study design was discussed with the institutional patient advisory group and representatives from patient organisations and was presented at the National Heart Failure Patient Health Care Professional Research Forum, receiving positive feedback. A senior heart failure expert was appointed as an independent trial data monitor, who reviewed study conduct and adverse events at scheduled meetings, was informed of serious adverse events as they occurred, and had the authority to terminate the trial if deemed necessary. Serious adverse events were reported to the trial sponsor within 24 h as per the protocol. The trial is registered on ClinicalTrials.gov, number NCT02859311, and the trial protocol is included in the appendix.

Inclusion criteria comprised a previous diagnosis of dilated cardiomyopathy with LVEF 40% or lower;^9^ absence of current symptoms of heart failure; current treatment with a loop diuretic, beta-blocker, angiotensin-converting enzyme (ACE) inhibitor, angiotensin receptor blocker (ARB), or mineralocorticoid receptor antagonist (MRA), or a combination of these drugs; a current LVEF of 50% or greater and a left ventricular end diastolic volume indexed to body surface area (LVEDVi) within the normal range on cardiovascular magnetic resonance (CMR);^10^ and plasma N-terminal pro-B-type natriuretic peptide (NT-pro-BNP) concentration less than 250 ng/L.

Exclusion criteria comprised uncontrolled hypertension (clinic blood pressure >160/100 mm Hg); valvular disease of moderate or greater severity; estimated glomerular filtration rate less than 30 mL/min per 1·73 m^2^; atrial, supraventricular, or ventricular arrhythmia requiring beta-blockade; pregnancy; angina; and age younger than 16 years.

Patients were identified from a registry, from clinics at the study centre and participant identification centres, and from patient organisations. Potential participants were invited for a screening visit and underwent comprehensive assessment. Imaging was done on a 3-Tesla scanner (Skyra, Siemens Healthcare; Erlangen, Germany*)* with a standardised protocol including late gadolinium enhancement imaging, T1 and extracellular volume (ECV) mapping, and strain assessment (appendix). Patients with contraindications to MRI underwent echocardiography with three-dimensional assessment of LV volumes (appendix). Blood was drawn for plasma NT-pro-BNP (Roche; Basel, Switzerland). Patients underwent a symptom-limited treadmill cardiopulmonary exercise test (CPET) with one of three ramp protocols (appendix). The protocol was selected on the basis of perceived exercise tolerance with the aim of completing the test within 8–12 min.^11^ Patients completed the Kansas City Cardiomyopathy Questionnaire (KCCQ) and a heart failure symptom assessment questionnaire (SAQ; appendix).

All patients provided informed consent and were enrolled if all inclusion criteria and none of the exclusion criteria were met. Diagnosis and possible cause of dilated cardiomyopathy was confirmed by consultant cardiologists with expertise in cardiomyopathy.

To gain insight into potential determinants of relapse, genetic sequencing was done with the TruSight Cardio Sequencing kit (Illumina, San Diego, CA, USA). Rare protein-altering variants were identified and interpreted as set out in guidelines. Further details about the sequencing are provided in the appendix.^12^ We focused on 12 genes with the most robust evidence for association with dilated cardiomyopathy: *TTN, DSP, MYH7, LMNA, TNNT2, TCAP, SCN5A, BAG3, TNNC1, VCL, TPM1*, and *RBM20*.^13^ A rare variant was defined as an ExAC filtering allele frequency less than 8·4 × 10^−5^.^14^ For *TTN,* only truncating variants that affect exons constitutively expressed in the heart were included.^15^

### Randomisation and masking

**Figure 1:**
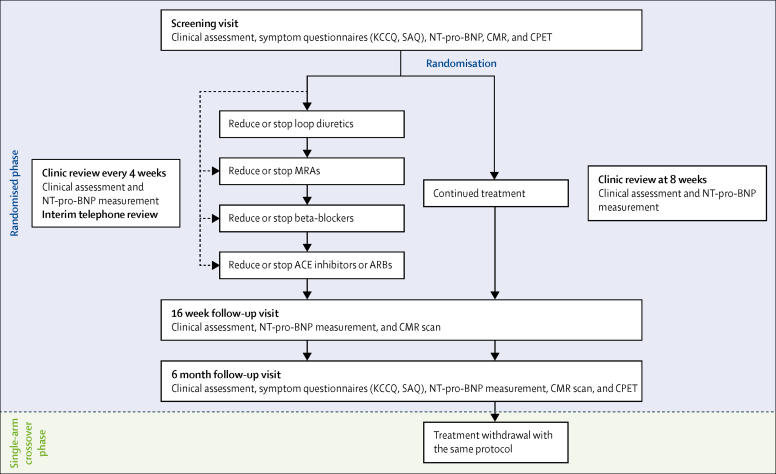
Flowchart of TRED-HF study design ACE=angiotensin converting enzyme. ARB=angiotensin receptor blocker. CMR=cardiovascular magnetic resonance. CPET=cardiopulmonary exercise test. KCCQ=Kansas City Cardiomyopathy Questionnaire. MRA=mineralocorticoid receptor antagonist. NT-pro-BNP=N-terminal pro-B-type natriuretic peptide. SAQ=symptom assessment questionnaire.

Patients were randomly assigned, by use of an online service, in random permuted blocks to supervised phased withdrawal or to continue treatment in a 1:1 allocation ratio, stratified by plasma NT-pro-BNP (0–50 ng/L, 50–125 ng/L, and 125–250 ng/L). This initial randomised phase of the study took place over 6 months (figure 1) and is outlined below. After 6 months, patients in the treatment withdrawal group completed the study and those initially assigned to continue treatment subsequently had phased withdrawal of treatment in the same way over the subsequent 6 months, as part of a single-arm crossover phase (figure 1). The study team, patients, and clinical teams knew which group the patient was assigned to. Patients were provided with labelled medications by the hospital pharmacy.

### Procedures

Patients randomly assigned to treatment withdrawal underwent supervised, step-wise reduction in pharmacological treatment over a maximum of 16 weeks (figure 1). Patients were reviewed every 2 weeks. Changes to medication were made following each review. Clinic visits and NT-pro-BNP measurements occurred at least every 4 weeks during withdrawal. Interim reviews took place via telephone if the patient remained asymptomatic between visits. Patients initially stopped or reduced the dose of loop diuretic, followed by MRA, beta-blocker, and finally ACE inhibitor or ARB. If the patient was taking the equivalent of, or less than 40 mg of frusemide or 50 mg of spironolactone, or 25% or less of the maximum recommended dose of beta-blocker, ACE inhibitor, or ARB, the medication was stopped. If the patient was taking a larger dose, this was reduced by 50% in a stepwise manner every 2 weeks. Further information about the algorithm is included in the appendix. Patients in the control group underwent clinical review with NT-pro-BNP measurement after 8 weeks.

At 16 weeks, all patients underwent a clinical review that included measurement of NT-pro-BNP concentration and a CMR scan to ascertain LV volumes and function. Patients had a further review at 6 months, which included a CMR scan to ascertain LV volumes and function, measurement of plasma NT-pro-BNP concentration, and a CPET by use of the same protocol as in the baseline visit. Patients completed the KCCQ and SAQ. Two patients with existing contraindications to MRI and two in whom it became contraindicated had three-dimensional echocardiography (appendix).

The progress of patients was reviewed at weekly meetings by a panel of investigators led by a consultant cardiologist. Treatments were re-established if patients fulfilled any of the primary endpoint criteria described below. Management of patients who did not meet the primary endpoint but had adverse events, such as episodes of arrhythmia, was discussed by the panel with their clinical teams. Decisions about restarting treatment following such episodes were made on an individual basis. Patients who developed hypertension without meeting the primary endpoint were managed with indapamide and amlodipine. Those with a blood pressure greater than 140/90 mm Hg who had diabetes or a 10-year cardiovascular risk of 20% or greater, and all those with a blood pressure greater than 160/100 mm Hg were started on hypertension treatment. All patients had 24 h access to a trial or on-call doctor who was available via telephone and provided medical advice and follow-up as required. Management of patients at the end of the trial was ascertained by their clinical teams in line with the protocol.

### Outcomes

The primary endpoint was a relapse of dilated cardiomyopathy within 6 months, defined by at least one of the following: a reduction in LVEF by more than 10% and to less than 50%; an increase in LVEDV by more than 10% and to higher than the normal range; a two-fold rise in baseline NT-pro-BNP concentration and to more than 400 ng/L; or clinical evidence of heart failure, based on signs and symptoms as adjudicated by the research team.

Secondary endpoints comprised a composite safety endpoint (cardiovascular mortality, major adverse cardiovascular events, and unplanned cardiovascular hospital admission) and the occurrence of sustained atrial or ventricular arrhythmias. Changes between baseline and follow-up, in LVEF, LVEDVi, plasma NT-pro-BNP concentration, left atrial volume indexed to body surface area (LAVi), KCCQ and SAQ scores (with lower scores on the KCCQ and higher scores on the SAQ indicating greater symptom burden), exercise time and peak oxygen consumption on CPET, heart rate, and blood pressure were also assessed.

Cardiac volumetric analysis was done by a Core Laboratory using CMR Tools (Cardiovascular Imaging Solutions, London, UK). Operators were masked to the treatment group and stage. Serial scans from each patient were analysed by the same operator. NT-pro-BNP was measured with the Elecsys immunoassay (Roche; Basel, Switzerland) throughout the study.

In May, 2017, the sponsor contacted the independent data safety monitor as the primary endpoint rate was higher than expected. It was deemed appropriate to complete the trial as planned given the absence of serious adverse events at this stage and the response to treatment re-initiation of those who met the primary endpoint.

### Statistical analysis

This was a pilot trial designed to assess the advisability and feasibility of doing a larger outcome trial, assuming that the pilot trial showed that the majority of patients could have treatment safely withdrawn without deterioration in cardiac function. The pilot trial was not prospectively powered to detect a difference in outcome. However, a retrospective power calculation showed that the sample size had 80% power to detect a difference in outcome, at a significance level of 5%, if the incidence of the primary endpoint in the intervention group was 26%, assuming there were no events in the control group.

Analysis was by intention to treat. Occurrence of the primary endpoint in the randomised phase of the study is graphically displayed per group with Kaplan-Meier survival plots and formally compared with the log-rank test. Baseline characteristics are presented according to assignment at randomisation and compared with the Mann-Whitney test for continuous variables or Fisher's exact test for categorical variables. Data are presented as median and IQR or as n (%).

Secondary outcome variables were compared between groups in the randomised phase by use of a regression model in which the outcome variable at 6 months was the response variable and the treatment indicator and outcome variable at baseline were explanatory variables (ie, analysis of covariance). Skewed variables (eg, NT-pro-BNP) were log-transformed to achieve a more normal distribution.

Since the number of patients in each group was small, we did a non-randomised analysis comparing secondary outcome variables immediately before treatment withdrawal versus 6 months later. This approach compared baseline versus 6-month values in patients randomly assigned to treatment withdrawal and 6-month versus 1-year variables for those randomly assigned to the control group who underwent treatment withdrawal during the crossover phase. Comparisons were made via paired *t* tests. In a prespecified exploratory analysis, we used Cox proportional hazards models to investigate whether any characteristics predicted occurrence of the primary outcome among patients who underwent treatment withdrawal, including those in the crossover phase.

A p value less than 0·05 was taken as significant throughout. Statistical analyses were done with Stata, version 15.1.

### Role of the funding source

The British Heart Foundation provided peer review of the grant proposal, and had no other role in study design, data collection, data analysis, data interpretation, or writing of the manuscript. The study received additional support from the Alexander Jansons Foundation, the Cardiovascular Research Centre and National Institute for Health Research Biomedical Research Unit at Royal Brompton Hospital and Imperial College London, the Imperial College Biomedical Research Centre, the Wellcome Trust, and Rosetrees Trust. They had no role in study design, data collection, data analysis, data interpretation, or writing of the manuscript. The corresponding author had full access to all the data in the study and had final responsibility for the decision to submit for publication.

## Results

**Figure 2:**
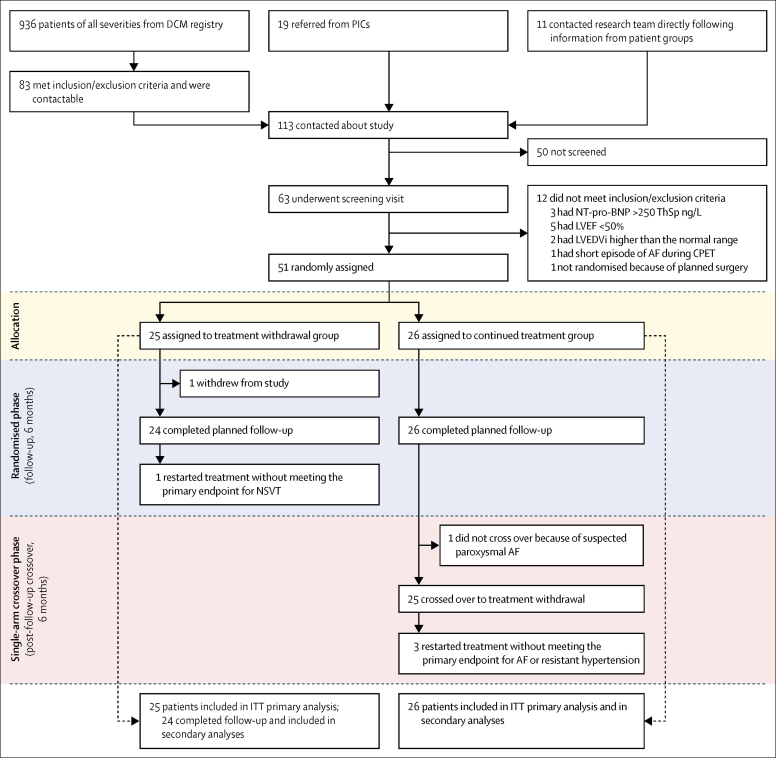
Trial profile One patient in the treatment withdrawal group who withdrew from the study was excluded from secondary analyses (table 3, table 4) because of absence of follow-up data. Therefore, 50 patients completed follow-up in the randomised phase. 49 patients completed follow-up after starting treatment withdrawal in the randomised and crossover phases (one patient did not cross over from the continued treatment group to begin treatment withdrawal). AF=atrial fibrillation. CPET=cardiopulmonary exercise test. DCM=dilated cardiomyopathy. ITT=intention-to-treat. LVEDVi=left ventricular end-diastolic volume indexed to body surface area. LVEF=left ventricular ejection fraction. NSVT=non-sustained ventricular tachycardia. NT-pro-BNP=N-terminal pro-B-type natriuretic peptide. PICs=participant identification centres.

Between April 21, 2016, and Aug 22, 2017, 51 of 63 screened patients met all inclusion criteria and none of the exclusion criteria and were randomly assigned and included in the intention-to-treat analysis (figure 2).

25 patients were randomly assigned to the treatment withdrawal group and 26 to the continued treatment group. Baseline characteristics were generally similar between the two groups (table 1). Among those randomly assigned to withdrawal versus those assigned to continued treatment, idiopathic dilated cardiomyopathy (80% *vs* 58%), previous atrial fibrillation (32% *vs* 15%), and previous unplanned heart failure admissions (72% *vs* 54%) were nominally more common. Overall, 34 (67%) patients were men and the median age was 55 years (IQR 45–64). The median LVEF at initial diagnosis was 25% (IQR 20–33) and the median time since diagnosis was 57 months (25–98) or 4·9 years (2·1–8·3) and time since recovery to LVEF greater than 50% was 24 months (6–43) or 2·0 years (0·7–3·5). At enrolment, all patients were in sinus rhythm, the median LVEF was 60% (IQR 55–64) and median plasma NT-pro-BNP concentration was 72 ng/L (39–135). At enrolment, all patients were taking an ACE inhibitor or ARB, 45 (88%) were taking a beta-blocker, and 24 (47%) an MRA, but only six (12%) were taking a loop diuretic. The six patients not taking a beta-blocker had been prescribed one at initial diagnosis but this treatment had been stopped before enrolment. Eight patients also previously on an MRA had this drug discontinued before enrolment. One patient in the control group had previous implantation of a cardiac resynchronisation device with a defibrillator for primary prevention purposes while one patient in the withdrawal group had an implantable cardioverter defibrillator in situ for secondary prevention purposes.

**Table 1:** Baseline characteristics of patients

	**Treatment withdrawal group (n=25)**	**Continued treatment group (n=26)**
**Demographics**		
Median age, years	54 (46 to 64)	56 (45 to 64)
Men	16 (64%)	18 (69%)
**Previous cardiovascular history**		
Time since initial DCM diagnosis, months	63 (36 to 112)	41 (20 to 91)
LVEF at initial diagnosis	28% (20 to 33)	25% (19 to 33)
Absolute improvement in LVEF	29% (23 to 36)	30% (25 to 38)
Time since LVEF >50%, months	28 (8 to 45)	20 (6 to 44)
Previous unplanned heart failure admission	18 (72%)	14 (54%)
Previous excess alcohol consumption	8 (32%)	9 (35%)
Previous atrial fibrillation	8 (32%)	4 (15%)
Previous hypertension	3 (12%)	1 (4%)
Diabetes	0 (0%)	1 (4%)
Smoker	0 (0%)	3 (12%)
**Cause**		
Idiopathic	20 (80%)	15 (58%)
Familial	3 (12%)	4 (15%)
Environmental insult	2 (8%)	7 (27%)
Truncating variant in *TTN*	7 (28%)	4 (15%)
**Medications at enrolment**		
ACE inhibitor or ARB	25 (100%)	26 (100%)
Beta-blocker	21 (84%)	24 (92%)
Mineralocorticoid receptor antagonist	12 (48%)	12 (46%)
Loop diuretic	3 (12%)	3 (12%)
**Clinical characteristics at enrolment**		
Body surface area, m^2^	2·1 (1·7 to 2·3)	2·0 (1·8 to 2·2)
Heart rate, bpm	62 (58 to 74)	70 (60 to 75)
Systolic blood pressure, mm Hg	123 (117 to 133)	127 (117 to 134)
Diastolic blood pressure, mm Hg	72 (68 to 80)	76 (70 to 80)
Left bundle branch block	3 (12%)	4 (15%)
QRS duration, ms	98 (85 to 108)	94 (88 to 111)
NT-pro-BNP, ng/L	72 (44 to 147)	75 (37 to 133)
**CMR variables at enrolment**		
LVEDVi, mL/m^2^	86 (66 to 91)	80 (70 to 91)
LVEF	62% (55 to 66)	60% (55 to 61)
LV mass index, g/m^2^	65 (53 to 76)	69 (62 to 76)
RVEDVi, mL/m^2^*	79 (66 to 92)	74 (62 to 92)
RVEF*	61% (57 to 64)	60% (54 to 65)
LAVi, mL/m^2^	41 (33 to 46)	41 (33 to 45)
Presence of late gadolinium enhancement*	10 (42)	10 (40)
Native T1 time, ms*	1293 (1253 to 1312)	1283 (1276 to 1328)
Extracellular volume*	25% (24 to 27)	26% (24 to 30)
Global radial strain†	0·30 (0·23 to 0·38)	0·25 (0·19 to 0·33)
Global circumferential strain†	−0·16 (−0·18 to −0·14)	−0·15 (−0·16 to −0·12)
Global longitudinal strain*	−0·14 (−0·15 to −0·13)	−0·13 (−0·16 to −0·11)
**CPET at enrolment**		
Peak VO_2_, mL/kg per min‡	29 (22 to 32)	26 (22 to 33)
Predicted peak VO_2_ %‡	95% (85 to 102)	90% (82 to 106)
Exercise time, s‡	571 (517 to 642)	577 (538 to 633)
**Symptom questionnaire scores**		
KCCQ, 0–100	97 (94 to 100)	94 (90 to 100)
SAQ, 0–185	11 (5 to 13)	10 (6 to 17)

Data are median (IQR) or n (%). Measurements at baseline screening visits. ACE=angiotensin-converting enzyme. ARB=angiotensin receptor blocker. bpm=beats per min. CMR=cardiovascular magnetic resonance. CPET=cardiopulmonary exercise test. DCM=dilated cardiomyopathy. KCCQ=Kansas City Cardiomyopathy Questionnaire. LAVi=left atrial volume indexed to body surface area. LV=left ventricular. LVEDVi=left ventricular end diastolic volume indexed to body surface area. LVEF=left ventricular ejection fraction. NT-pro-BNP=N-terminal pro-B-type natriuretic peptide. RVEDVi=right ventricular end diastolic volume indexed to body surface area. RVEF=right ventricular ejection fraction. SAQ=symptom assessment questionnaire. VO_2_=oxygen consumption.

* Two patients did not undergo CMR at baseline because of contraindications.

† In addition to the two patients who did not undergo CMR, global circumferential and radial strain could not be calculated from images available for a third patient.

‡ Four patients did not undergo CPET at baseline because of musculoskeletal pain or injury.

35 (69%) patients had idiopathic dilated cardiomyopathy, seven (14%) had familial dilated cardiomyopathy, and nine (18%) had dilated cardiomyopathy secondary to a trigger including previous excess alcohol consumption, pregnancy, remote anthracycline administration, hyperthyroidism, and a previous episode of myocarditis. 39 (76%) patients (20 randomly assigned to treatment withdrawal and 19 to continue treatment) had coronary angiography with no evidence of obstructive disease (defined as a stenosis >50% in a major coronary artery) and a further four (8%; three randomly assigned to treatment withdrawal and one to continue treatment) had no evidence of ischaemia on nuclear or magnetic resonance stress imaging. Of the remaining eight (16%) patients (two randomly assigned to treatment withdrawal and six to continue treatment), seven (one randomly asssigned to treatment withdrawal and six to continue treatment) were younger than 50 years, none had evidence of myocardial infarction on imaging, and all were considered to have a low probability of coronary disease.

11 (22%) of 51 patients had a rare, constitutively expressed, truncating variant in *TTN* that was classified as being likely pathogenic (appendix). No other pathogenic or likely pathogenic variants were identified in genes associated with dilated cardiomyopathy (appendix).

**Figure 3:**
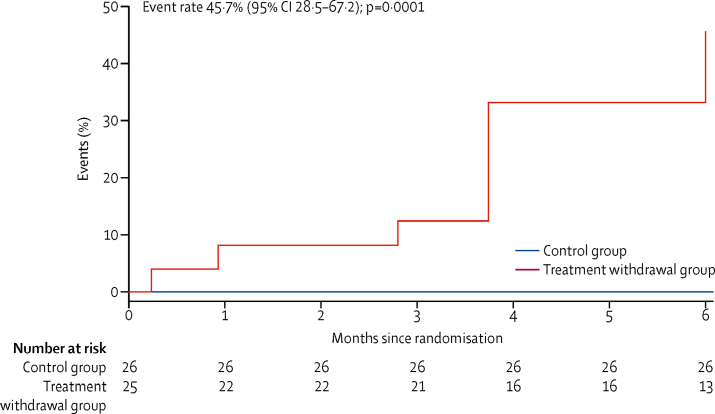
Kaplan-Meier curve of time to primary endpoint in randomised phase, according to treatment group One patient dropped out at 7 days.

Of the 25 patients initially randomly assigned to phased withdrawal of treatment, 11 (44%) met the primary endpoint criteria for relapse within 6 months compared with none of those assigned to continued treatment (figure 3). The Kaplan-Meier estimate of the event rate at 6 months in the withdrawal group was 45·7% (95% CI 28·5–67·2; p=0·0001). One patient randomly assigned to withdrawal dropped out of the study after enrolment following further discussion with their cardiologist. After 6 months, in the single-arm crossover phase of the study, 25 (96%) of 26 patients initially assigned to continued treatment underwent phased withdrawal of treatment, nine of whom met the criteria for relapse during the following 6 months (Kaplan-Meier estimate of event rate 36·0% [95% CI 20·6–57·8]; appendix). One patient did not undergo treatment withdrawal because of symptoms indicative of atrial fibrillation while on treatment during the first 6 months. Therefore, of the 50 patients who began withdrawal, 20 (40%) relapsed during the study period. 13 (26%) patients relapsed within 16 weeks of beginning withdrawal (eight in the treatment withdrawal group and five in the crossover phase), all of whom relapsed within 8 weeks of taking their last medication. Additionally, four patients restarted heart failure treatment without meeting the primary endpoint, including two for hypertension refractory to treatment with other drugs (in the crossover phase), one following an episode of atrial fibrillation (in the crossover phase), and one following an episode of non-sustained ventricular tachycardia (in the treatment withdrawal group; figure 2). Therefore, only 25 (50%) of 50 patients successfully completed 6 months of follow-up without re-initiation of treatment.

**Figure 4:**
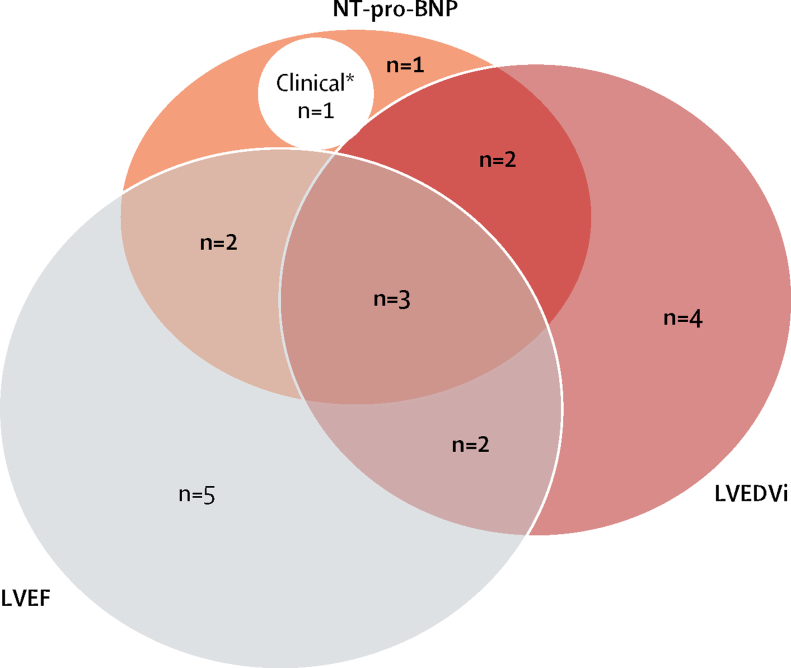
Venn diagram showing components contributing to primary endpoint definition Numbers of patients with each combination of endpoints included. LVEDVi=left ventricular end-diastolic volume indexed to body surface area. LVEF=left ventricular ejection fraction. NT-pro-BNP=N-terminal pro-B-type natriuretic peptide. *Refers to one patient who developed peripheral oedema.

Of the 20 patients who met the primary endpoint, ten fulfilled more than one criterion for relapse. 12 (60%) met the LVEF criterion for relapse, 11 (55%) met the LVEDVi criterion, nine (45%) met the NT-pro-BNP criterion, and one (5%) developed peripheral oedema (figure 4; appendix). Two patients developed shortness of breath upon moderate exertion (New York Heart Association Class [NYHA] II). Nine of ten patients who only met one endpoint criterion also had deterioration in at least one other variable that did not reach the prespecified threshold. The remaining patient had a reduction in LVEF from 52% at baseline to 41% at 16 weeks.

No deaths, unplanned hospital admissions for heart failure, or major adverse cardiovascular events were reported in either group. Three serious adverse events were reported in the treatment withdrawal group: hospital admissions for urinary sepsis, non-cardiac chest pain, and an elective procedure for a pre-existing condition. Neither patient with an implantable cardioverter-defibrillator received treatment from the device. Three patients developed atrial fibrillation during treatment withdrawal, two of whom met the primary endpoint.

All patients who met the primary endpoint subsequently restarted treatment. At the next follow-up, none of the patients had symptoms of heart failure (NYHA class I) and 17 (85%) of 20 had LVEF greater than 50%. Two patients had improvements in LVEF to between 45–50% and one had a reduction in LVEF from 48% to 43%, prompting intensification of medical therapy. In prespecified exploratory analyses to identify predictors of the primary endpoint, the following baseline characteristics were associated with increased risk of relapse in univariable analyses: advancing age (p=0·0309), prescription of an MRA before withdrawal (p=0·0042), prescription of more than two heart failure medications (p=0·0040), increased NT-pro-BNP concentration (p=0·0161), and decreased peak global radial strain (p=0·0177; table 2). Patients with lower LVEF at original diagnosis and lower peak oxygen consumption on exercise tests seemed to have higher rates of relapse than those who did not, but these results were not significant. Familial dilated cardiomyopathy, presence of a truncating variant in *TTN,* LVEF at enrolment, time since original dilated cardiomyopathy diagnosis, and presence of late gadolinium enhancement were not significantly associated with the primary endpoint. However, the study was not adequately powered to detect such associations. Characteristics of patients who relapsed and those who did not are presented in the appendix.

**Table 2:** Hazard ratios for primary outcome by patient characteristics among those who underwent treatment withdrawal

		**Patients**	**Events**	**Hazard ratio (95% CI)**	**p value**
**Demographics**					
Age (per 10 years)		50	20	1·6 (1·0 to 2·4)	0·0309
Women		17	9	1·0 (ref)	0·3190
Men		33	11	0·64 (0·26 to 1·53)	..
**Previous diagnosis and history**					
Time since initial diagnosis, years		50	20	1·1 (0·71 to 1·7)	0·6587
LVEF at initial diagnosis, %		50	20	0·73 (0·48 to 1·12)	0·148222
Previous atrial fibrillation					
	No	39	17	1·0 (ref)	0·4897
	Yes	11	3	0·66 (0·19 to 2·26)	..
Previous heart failure admission					
	No	19	9	1·0 (ref)	0·5109
	Yes	31	11	0·74 (0·31 to 1·79)	..
**Causal factors**					
Cause of DCM					
	Idiopathic	34	13	1·0 (ref)	0·9487
	Familial	7	3	1·2 (0·4 to 4·3)	..
	Environmental insult	9	4	1·0 (0·3 to 3·2)	..
Truncating variant in *TTN*					
	No	39	16	1·00 (ref)	0·9765
	Yes	11	4	0·98 (0·33 to 2·94)	..
**Medications at baseline**					
ACE inhibitor or ARB		50	20	NA	NA
Not on beta-blocker (ref)		6	1	1·0 (ref)	0·1708
Beta-blocker		44	19	3·2 (0·4 to 24·1)	..
Not on MRA (ref)		26	5	1·0 (ref)	0·0042
MRA		24	15	3·9 (1·4 to 10·8)	..
Not on loop diuretic (ref)		44	16	1·0 (ref)	0·1575
Loop diuretic		6	4	2·4 (0·8 to 7·1)	..
Number of heart failure medications					
	≤2 drugs	26	5	1·0 (ref)	0·0040
	3 drugs	19	12	3·7 (1·3 to 10·6)	..
	4 drugs	5	3	4·8 (1·1 to 20·2)	..
**Clinical characteristics at enrolment**					
Heart rate, bpm		50	20	1·2 (0·8 to 1·9)	0·3120
Systolic blood pressure, mm Hg		50	20	0·81 (0·51 to 1·31)	0·3959
Diastolic blood pressure, mm Hg		50	20	0·91 (0·58 to 1·43)	0·6802
LBBB					
	No	43	17	1·0 (ref)	0·9042
	Yes	7	3	1·1 (0·3 to 3·7)	..
QRS duration, ms		50	20	1·0 (0·7 to 1·6)	0·8713
Log NT-pro-BNP, ng/L		50	20	1·8 (1·1 to 2·8)	0·0161
**CMR measurements at enrolment**					
LVEF, %		50	20	0·81 (0·51 to 1·28)	0·3681
LVEDVi, mL/m^2^*		50	20	1·1 (0·7 to 1·8)	0·5838
LAVi, mL/m^2^*		50	20	1·0 (0·7 to 1·5)	0·9922
Presence of LGE*					
	No	28*	11	1·0 (ref)	0·9669
	Yes	20	8	0·98 (0·39 to 2·44)	..
Global radial strain†		47†	19	0·55 (0·34 to 0·90)	0·0177
Global circumferential strain†		47†	19	1·3 (0·8 to 2·0)	0·3365
Global longitudinal strain*		48*	19	1·2 (0·8 to 1·8)	0·5080
Native T1, ms*		48*	19	0·98 (0·63 to 1·51)	0·9163
ECV, %*		48*	19	1·2 (0·80 to 1·79)	0·3933
**CPET measurements at enrolment**					
Peak VO_2_, mL/kg per min‡		47‡	19	0·63 (0·39 to 1·04)	0·0703
Predicted peak VO_2_, %‡		47‡	19	1·1 (0·7 to 1·7)	0·7162

Data are n, unless otherwise stated. Univariable proportional hazard modelling. Timepoint for characteristics taken from the beginning of treatment withdrawal (at baseline for those randomly assigned to treatment withdrawal and at the start of the single-arm crossover phase for those initially randomly assigned to continue treatment). Hazard ratios for continuous variables presented per 1 SD higher, apart from age which is presented per 10 years (see appendix). Hazard ratios for categorical variables presented with the reference group indicated in the table at each point. ACE=angiotensin-converting enzyme. ARB=angiotensin receptor blocker. bpm=beats per min. CMR=cardiovascular magnetic resonance. CPET=cardiopulmonary exercise test. DCM=dilated cardiomyopathy. ECV=extracellular volume. LAVi=left atrial volume indexed to body surface area. LBBB=left bundle branch block. LGE=late gadolinium enhancement. LVEDVi=left ventricular end diastolic volume indexed to body surface area. LVEF=left ventricular ejection fraction. MRA=mineralocorticoid receptor antagonist. NA=not available. NT-pro-BNP=N-terminal pro-B-type natriuretic peptide. VO_2_=oxygen consumption.

* Two patients did not undergo CMR at start of treatment withdrawal because of contraindications.

† In addition to the two patients who did not undergo CMR, global circumferential and radial strain could not be calculated from images available for a third patient.

‡ Three patients did not undergo CPET at the start of treatment withdrawal because of pain or injury.

**Figure 5:**
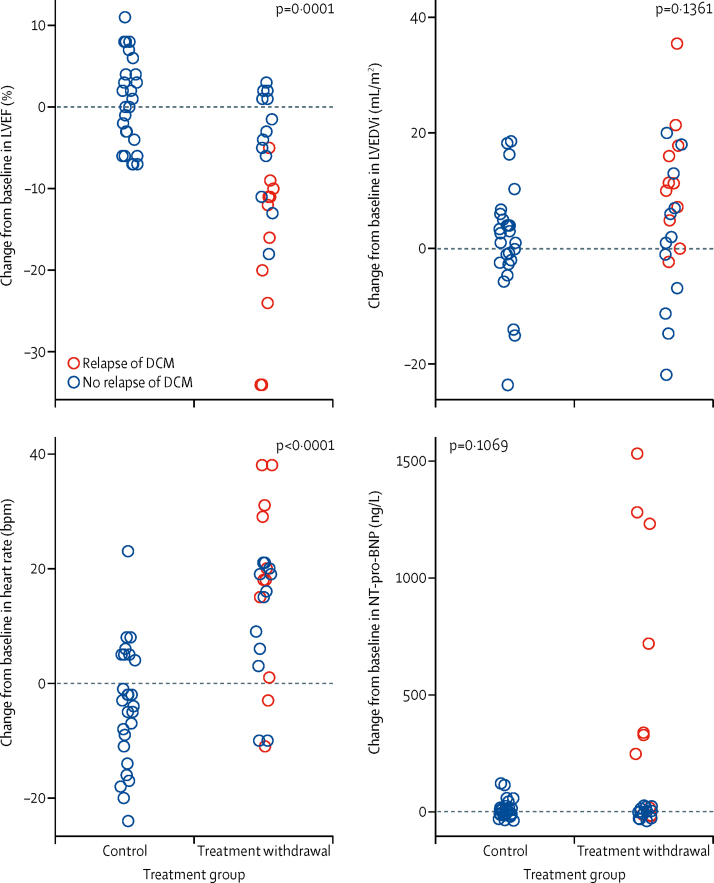
Change in secondary endpoint variables between baseline and follow-up in the randomised phase of the study, based on treatment group Each circle represents one patient. bpm=beats per min. DCM=dilated cardiomyopathy. LVEDVi=left ventricular end-diastolic volume indexed to body surface area. LVEF=left ventricular ejection fraction. NT-pro-BNP=N-terminal pro-B-type natriuretic peptide.

With regard to secondary endpoints between groups in the randomised phase, treatment withdrawal was associated with a significant decline in LVEF, a significant increase in heart rate and diastolic blood pressure, and a significant decline in KCCQ score (table 3, figure 5). The effect of treatment withdrawal on other secondary variables was not significant; however, we observed non-significant increases in LVEDVi, systolic blood pressure, and log NT-pro-BNP (table 3).

**Table 3:** Secondary outcomes at baseline and at 6-month follow-up by treatment group in the randomised phase

	**Mean (SD) in continued treatment group**	**Mean (SD) in treatment withdrawal group**	**Estimated mean effect of treatment withdrawal on 6-month values (95% CI)**	**p value**
**LVEF, % (n=50)**				
Baseline	59 (5)	61 (6)	..	..
6 months	59 (59)	51 (51)	−9·5 (−14·0 to −4·9)	0·0001
LVEDVi, mL/m^2^ (n=49*)				
Baseline	80 (13)	79 (14)	..	..
6 months	81 (81)	84 (84)	4·7 (−1·5 to 11·0)	0·1361
LAVi, mL/m^2^ (n=49*)				
Baseline	41 (9)	40 (8)	..	..
6 months	42 (42)	41 (41)	0·5 (−4·2 to 5·2)	0·8217
**Heart rate, bpm (n=50)**				
Baseline	70 (10)	66 (13)	..	..
6 months	66 (66)	80 (80)	15·4 (10·0 to 20·9)	<0·0001
**Systolic blood pressure, mm Hg (n=50)**				
Baseline	125 (12)	123 (12)	..	..
6 months	125 (125)	133 (133)	6·6 (−0·1 to 13·4)	0·0547
**Diastolic blood pressure, mm Hg (n=50)**				
Baseline	74 (8)	73 (10)	..	..
6 months	73 (73)	79 (79)	7·0 (1·9 to 12·1)	0·0083
**Log NT-pro-BNP concentration, ng/L (n=50)**				
Baseline	4·2 (0·8)	4·2 (0·7)	..	..
6 months	4·3 (4·3)	4·7 (4·7)	0·3 (−0·1 to 0·7)	0·1069
**Peak VO**_2_**, mL/kg per min (n=43**†**)**				
Baseline	27 (7)	28 (7)	..	..
6 months	27 (27)	26 (26)	−1·2 (−3·9 to 1·6)	0·4020
**Exercise time, s (n=43**†**)**				
Baseline	580 (77)	582 (73)	..	..
6 months	579 (579)	569 (569)	−3·4 (−36·0 to 29·2)	0·8344
**KCCQ, 0–100 (n=49**‡**)**				
Baseline	93 (8)	96 (4)	..	..
6 months	94 (94)	91 (91)	−5·1 (−9·9 to −0·4)	0·0354
**SAQ, 0–100 (n=49**‡**)**				
Baseline	2·3 (0·7)	2·1 (0·7)	..	..
6 months	2·2 (2·2)	2·3 (2·3)	0·3 (−0·1 to 0·6)	0·1483

Measurements at baseline and follow-up in the randomised phase based on assignment at randomisation. bpm=beats per min. KCCQ=Kansas City Cardiomyopathy Questionnaire. LAVi=left atrial volume indexed to body surface area. LVEDVi=left ventricular end diastolic volume indexed to body surface area. LVEF=left ventricular ejection fraction. NT-pro-BNP=N-terminal pro-B-type natriuretic peptide. SAQ=symptom assessment questionnaire. VO_2_=oxygen consumption.

* One patient did not have LVEDVi and LAVi data available at follow-up because of a new contraindication to cardiovascular magnetic resonance (three-dimensional echocardiography used for LVEF follow-up).

† Seven patients were unable to complete cardiopulmonary exercise test at either baseline and follow-up because of musculoskeletal pain or injury.

‡ One patient did not complete questionnaires at follow-up.

In the non-randomised comparison of baseline versus follow-up variables for all patients who had treatment withdrawal attempted and completed follow-up (n=49), we observed a significant reduction in LVEF and a significant increase in LVEDVi, log NT-pro-BNP, heart rate, systolic blood pressure, and diastolic blood pressure (table 4). In the analysis of patients who did not meet the primary endpoint, we observed a significant decline in LVEF and a significant increase in heart rate, systolic blood pressure, and diastolic blood pressure (table 4).

**Table 4:** Non-randomised comparison of secondary outcomes before and after treatment withdrawal for all patients, according to occurrence of primary endpoint

	**Overall**			**Primary endpoint met**			**Primary endpoint not met**		
	Patients	Estimated mean change between baseline and 6 months (95% CI)	p value	Patients	Estimated mean change between baseline and 6 months (95% CI)	p value	Patients	Estimated mean change between baseline and 6 months (95% CI)	p value
LVEF, %	49	−6·9 (−9·6 to −4·3)	<0·0001	20	−12·0 (−16·6 to −7·4)	0·0001	29	−3·5 (−5·8 to −1·1)	0·0190
LVEDVi, mL/m^2^	47*	6·5 (3·1 to 9·8)	0·0003	20	11·8 (8·2 to 15·3)	<0·0001	27*	2·5 (−2·0 to 7·0)	0·2107
LAVi, mL/m^2^	47*	2·0 (−0·6 to 4·6)	0·1224	20	6·6 (3·3 to 9·9)	0·0009	27*	−1·4 (−4·5 to 1·7)	0·3702
Heart rate, bpm	49	13·2 (9·3 to 17·1)	<0·0001	20	16·4 (9·1 to 23·6)	0·0003	29	11·7 (7·9 to 15·6)	<0·0001
Systolic blood pressure, mm Hg	49	8·7 (4·6 to 12·9)	0·0001	20	8·9 (2·3 to 15·4)	0·0101	29	8·7 (3·4 to 13·9)	0·0020
Diastolic blood pressure, mm Hg	49	6·7 (3·2 to 10·1)	0·0003	20	6·4 (1·7 to 11·0)	<0·0001	29	6·9 (2·2 to 11·5)	0·0033
Log NT-pro-BNP, ng/L	49	0·3 (0·0 to 0·6)	0·0246	20	0·4 (0·2 to 0·6)	0·0022	29	0·0 (−0·1 to 0·05)	0·4276
VO_2_ max (mL/kg per min)	41†	−0·7 (−2·1 to 0·7)	0·3294	17†	−1·5 (−3·5 to 0·4)	0·1476	24†	0·0 (−1·9 to 2·0)	0·9737
Exercise time (s)	41†	−0·6 (−14·9 to 13·8)	0·9376	17†	−19·7 (−40·9 to 1·5)	0·0873	24†	12·0 (−4·4 to 28·3)	0·1646
KCCQ, 0–100	49	−2·2 (−4·7 to 0·3)	0·0777	20	−3·9 (−7·7 to −0·11)	0·0582	29	−1·2 (−4·2 to 1·8)	0·4480
SAQ, 0–100	49	0·1 (−0·1 to 0·3)	0·3782	20	1·5 (−2·9 to 5·9)	0·5110	29	0·77 (−1·9 to 3·4)	0·5754

Measurements taken at the start of treatment withdrawal (at baseline for those randomly assigned to treatment withdrawal and at the start of the single-arm crossover phase for those initially randomly assigned to continue treatment) and follow-up. A maximum of 49 patients completed follow-up. bpm=beats per min. KCCQ=Kansas City Cardiomyopathy Questionnaire. LAVi=left atrial volume indexed to body surface area. LVEDVi=left ventricular end diastolic volume indexed to body surface area. LVEF=left ventricular ejection fraction. NT-pro-BNP=N-terminal pro-B-type natriuretic peptide. SAQ=symptom assessment questionnaire. VO_2_ max=maximum oxygen consumption.

* Two patients had absent LVEDVi and LAVi at follow-up because of new contraindication to cardiovascular magnetic resonance; three-dimensional echocardiography used for LVEF follow-up.

† Eight patients were unable to complete the cardiopulmonary exercise test because of musculoskeletal pain or injury.

Of the 50 patients who began treatment withdrawal, eight (16%) had a potentially reversible insult, including four with previous excess alcohol consumption, two with peripartum presentation, one with previous myocarditis, and one with hyperthyroidism. Of these patients, three (38%) met the primary endpoint, including two with previous excess alcohol consumption and one with peripartum presentation. Among these eight patients, mean LVEF at enrolment was 59% (SD 6·1) and the mean LVEF at follow-up was 55% (7·7; mean difference −4·6% [95% CI −13·1 to 3·8]; p=0·2333).

16 (32%) patients (nine in the treatment withdrawal group, seven in the crossover phase) completed treatment withdrawal, remained asymptomatic, and either finished the study with LVEF in the normal range or had a 3% or lower absolute reduction in LVEF (the expected interstudy variability^16^) or an increase in LVEF. The characteristics of these patients are included in the appendix.

## Discussion

To the best of our knowledge, this study is the first prospective randomised trial to investigate withdrawal of heart failure treatment in patients deemed to have recovered dilated cardiomyopathy. In this pilot study, treatment was withdrawn successfully in only 50% of patients, while 40% had a relapse of their dilated cardiomyopathy within 6 months. Potentially, more patients might have relapsed with a longer duration of treatment withdrawal. This finding suggests that, for many patients, improvement in cardiac function following treatment does not reflect full and sustained recovery but rather reflects remission, which requires at least some treatment to be maintained. Withdrawal of treatment should therefore not be attempted routinely in these patients. Further research should aim to identify variables that discriminate remission from permanent recovery. This research could enable safe withdrawal of treatment in some subgroups. Given the speed of deterioration, with most patients relapsing within 8 weeks of their last medication, these findings also provide guidance about how to monitor patients if treatment withdrawal is attempted, either at the patient's request, because of side-effects, or because the patient is considering pregnancy.

Secondary analyses of the randomised groups showed worsening KCCQ scores, a substantial reduction in LVEF, and non-significant increases in NT-pro-BNP and LV volumes. These findings were supported by the non-randomised comparison of baseline and follow-up variables among patients who had treatment withdrawal. Therefore, deterioration in LVEF after treatment withdrawal is not simply an imaging artifact but reflects disease recurrence. Analyses of patients who had withdrawal but did not meet the primary endpoint showed that these patients also had an overall reduction in LVEF. Our data also show that deterioration in LVEF often predated a rise in natriuretic peptides. Reproducible imaging, such as MRI, therefore appears to be important if withdrawal is considered under certain circumstances.

Patient safety was a priority. To identify those with the greatest degree of recovery, we only included asymptomatic patients with normal LV volumes and LVEF greater than 50% and excluded patients with clearly elevated plasma concentrations of NT-pro-BNP. Participants were monitored closely and treatment was restarted as soon as any criteria for the endpoint were met or if there was another reason to resume treatment, such as atrial fibrillation. The absence of major adverse cardiovascular events and unplanned hospital admissions for heart failure reflects the early detection of deterioration and restarting of medications before decompensation. It would have been unethical to wait until decompensation was clinically overt after detecting deteriorating cardiac function before reinstating treatment. We focused on patients with dilated cardiomyopathy as these patients are most likely to recover. Our data should be extrapolated with caution to patients with recovered left ventricular function secondary to ischaemic heart disease or hypertension. In ischaemic heart disease, ACE inhibitors and beta-blockers can be given to reduce further coronary events as well as morbidity and mortality related to heart failure. The same treatment is also given to control blood pressure in patients with hypertensive disease. Patients with recovered dilated cardiomyopathy also have a low prevalence of other comorbidities that might require pharmacological therapy, as indicated by our cohort and previous studies.^3^

Previous studies examining treatment withdrawal have been done in heterogeneous populations. Early studies were done in patients with reduced LVEF and symptoms of heart failure, most of whom had ischaemic heart disease.^17^, ^18^, ^19^, ^20^ More contemporary studies in patients with dilated cardiomyopathy have been retrospective and done in poorly characterised populations, providing conflicting results.^21^, ^22^ Our trial therefore provides the first prospective information about the risks of withdrawing treatment in patients with recovered dilated cardiomyopathy.

Moon and colleagues^21^ retrospectively investigated 42 patients with dilated cardiomyopathy whose LVEF had improved to greater than 40%. Seven patients discontinued treatment, five of whom subsequently had a reduction in LVEF at a median follow-up of 32 months. However, most patients who deteriorated had LVEF less than 50% and LV dilatation. Conversely, Amos and colleagues^22^ studied 22 patients with peripartum cardiomyopathy whose LVEF had improved to greater than 50%. Ten subsequently stopped either an ACE inhibitor or beta-blocker and five stopped both medications. None of the patients had a deterioration in LVEF over a median follow-up of 29 months. Differences in outcome might be related to the cause of dilated cardiomyopathy, but it is becoming increasingly clear that overlap exists between what have traditionally been viewed as separate acquired and genetic conditions.^23^, ^24^ For example, a common genetic predisposition appears to exist for many patients with idiopathic and peripartum dilated cardiomyopathy.^23^ Although identification of a trigger might improve the prediction of outcome, triggers can occur many years before presentation and their significance at the time of diagnosis is often unclear. Because of the overlap between causes, the absence of data about any subgroup and to produce a sample that was representative of the real-world population, we chose to include all causes of dilated cardiomyopathy. Similar to registries, most patients in this trial were labelled as having idiopathic disease.^25^ Considering the complexity of defining the cause, it is not surprising that we found no association between the perceived cause and relapse in this trial, although the sample size was not large enough to detect more subtle associations.

Being able to distinguish between patients who have complete recovery as opposed to remission is an important goal. Traditional practice has focused on the use of LVEF and LV volumes to grade recovery. However, within this small trial these variables were poor at predicting relapse. Whether the rate of relapse differs according to sex and age is unclear and deserves further work. It would be reasonable to believe that patients with genetic disease would be at increased risk of relapse. However, the relapse rate was not clearly greater in those with familial disease or a truncating variant in *TTN*. Previous reports have shown the potential for reverse remodelling in patients with a truncating variant in *TTN*,^15^, ^26^ which is consistent with the high prevalence of a truncating variant in *TTN* in our cohort. Imaging and circulating biomarkers offer hope for improving disease characterisation. Global radial strain and NT-pro-BNP concentration were associated with the risk of relapse in exploratory analyses in this small sample. Whether these analyses are reproducible and able to reliably distinguish between recovery and remission requires further work. Identification of multiple characteristics that reflect different aspects of myocyte function might be needed.

Although this study is, to our knowledge, the only and therefore largest randomised trial investigating treatment withdrawal for recovered dilated cardiomyopathy, the power to examine the association between baseline characteristics and relapse was restricted by the number of participants. The study might also have been too small to identify differences in variables such as NT-pro-BNP, which appeared to lag behind the reduction in LVEF. Although there were differences in some baseline characteristics between groups, these were small in absolute numbers and none of these variables was associated with the primary outcome. The single-arm crossover phase adds support for a consistent effect size across the entire population. The study was done in a single centre. Although some single-centre studies might be susceptible to selection bias, patients in this study were recruited from referral sites covering a large, diverse population. The baseline characteristics suggest that the cohort encompassed the heterogeneity of patients with dilated cardiomyopathy. A minimum time with LV dysfunction before recovery was not specified. However, diagnosis of dilated cardiomyopathy was confirmed by experienced clinicians and fulfilled diagnostic criteria.

Although imaging and laboratory investigations were masked to the assigned group to minimise bias in objective measures, patients and clinicians knew to which group patients were assigned. Patients might have been biased in favour of treatment withdrawal and attributed side-effects to medications they did not wish to take or denied the presence of mild symptoms. It is also possible that treatment withdrawal provoked anxiety. These possibilities should be considered when interpreting the patient-reported deterioration in symptoms. Patients in the treatment withdrawal group also underwent more frequent follow-up than did those in the continued treatment group. Although this aspect of the study design could be a source of bias, only two of 20 events were detected, without development of symptoms, at visits before 16 weeks. We attempted to withdraw all treatment for heart failure. Many patients had already stopped diuretics. No patients were taking angiotensin receptor-neprilysin inhibitors as almost all were diagnosed before introduction of these drugs and showed favourable response to previous conventional treatment.^27^

The rise in heart rate associated with treatment withdrawal, which was most marked in those who relapsed, suggests that the withdrawal of beta-blockers might be an important factor. However, one patient not on a beta-blocker at baseline relapsed following treatment withdrawal. Heart rate is closely linked to prognosis in patients with heart failure and sinus rhythm.^28^, ^29^ Whether the ongoing benefit of beta-blockers in this population is solely related to heart rate suppression deserves further consideration. Future work should explore which component of treatment is most important in preventing relapse. The impact of a change in cardiac function following treatment withdrawal on future compliance with medication is another worthwhile topic.

In conclusion, in this pilot study, withdrawal of pharmacological heart failure treatment in patients with recovered dilated cardiomyopathy was associated with relapse in 40% of cases. This finding suggests that complete withdrawal of treatment should not usually be attempted in such patients. Future work could identify patient subgroups who have permanent recovery of myocardial function for whom withdrawal is safe or for whom only some medications need to be continued in the long term.

## Data sharing
