# Supplementary material for: Withdrawal of pharmacological treatment for heart failure in patients with recovered dilated cardiomyopathy (TRED-HF): an open-label, pilot, randomised trial
Source: Lancet. 2019 Jan 5;393(10166):61–73. doi: 10.1016/S0140-6736(18)32484-X (PMC6319251; doi:10.1016/S0140-6736(18)32484-X)
Supplement: Supplementary appendix [file mmc1.pdf]

THE LANCET

Supplementary appendix

This appendix formed part of the original submission and has been peer reviewed. We post it as supplied by the authors.

Supplement to: Halliday BP, Wassall R, Lota AS, et al. Withdrawal of pharmacological treatment for heart failure in patients with recovered dilated cardiomyopathy (TRED-HF): an open-label, pilot, randomised trial. *Lancet* 2018; published online Nov 11. http://dx.doi.org/10.1016/S0140-6736(18)32484-X.

Supplementary Appendix

1. Cardiovascular Magnetic Resonance Protocol

CMR was performed using a standardised protocol on a 3 Tesla scanner (*Skyra, Siemens, Erlangen, Germany*). Localiser images were acquired using HASTE imaging. Following this resting long- and short-axis cine images were taken using breath-hold SSFP imaging. In order to calculate myocardial strain, images were acquired using displacement encoding with stimulated echoes (DENSE) in 2- and 4-chamber planes and in a short-axis plane at mid-ventricular level.[1, 2] Native T1 maps were also acquired at basal- and mid-ventricular level in short-axis planes, using a breath-hold 5-3-3 modified Look-Locker inversion recovery (MOLLI) sequence. Two maps were acquired in each plane. Post-contrast T1 maps were also acquired, 15 minutes after the administration of gadolinium, in identical locations to the pre-contrast maps, using the same breath-hold 5-3-3 MOLLI sequence. Immediately before and after the acquisition of post-contrast T1 maps, LGE imaging was performed using a phase sensitive inversion recovery sequence, starting around 10 minutes after the intravenous administration of gadobutrol (0.1mmol/kg). Images were acquired in identical short- and long-axis planes to cine imaging.

Image Analysis

Volumetric analysis was carried out using CMR Tools (*Cardiovascular Imaging Solutions, London)*. The biplane area length method was used to calculate left atrial volume.[3] The presence of LGE was determined by an independent operator and judged to be present if seen in two orthogonal planes and in two-phase encoding directions. T1 maps were analysed using CMR Tools (*Cardiovascular Imaging Solutions, London).* Raw images were initially inspected for artefact secondary to cardiac or respiratory motion. Those deemed satisfactory were analysed by drawing a crescent-shaped region of interest in the middle third of the septum, in order to avoid contamination of the myocardial signal with that of the blood pool. On the same image, an additional circular region of interest was drawn in the blood pool, avoiding trabeculae and papillary muscles. The software was then used to calculate the T1 values from the specified regions. Using the native and post-contrast T1 values and the haematocrit value, measured immediately prior to the scan, the extracellular volume (ECV) fraction was calculated.[4] Myocardial strain was calculated from DENSE data using semi-automated software on Matlab (*Mathworks, Natick, USA*), developed by the University of Virginia.[5] For long-axis images, a contour was placed in the mid-myocardium of the left ventricle in peak systole or diastole. For short-axis images, a region of interest was defined, including the endocardial and epicardial borders of the LV. The contours were then propagated to the remaining phases of the cardiac cycle using motion-guided segmentation. Minor manual adjustments were made when necessary. Regional polar strain time curves were generated for radial and circumferential strain and contour strain/time curves for longitudinal strain. From this data, peak global longitudinal, circumferential and radial strain were calculated.

2. Echocardiography Protocol

Patients unable to undergo CMR due to contraindications, such as previous device implantation, underwent comprehensive echocardiographic assessment. Studies were performed by the same operator using a commercially available system (*iE33, Philips Healthcare, Best, The Netherlands*) with a 3.5 MHz transducer for 2D study and with a X5-1 transducer for 3D study. Measurements were made according to current guidelines.[6, 7] LV volumes were traced manually at end-diastole and end-systole in apical 4- and 2-chamber views. LVEF was calculated using the modified Simpson's biplane method. LV mass was calculated using the corrected American Society of Echocardiography method. LA volume was measured using the biplane method in 4- and 2-chamber. To assess LV diastolic function, E/A ratio and deceleration time were calculated. In addition, tissue Doppler indices were measured in the apical 4-chamber view. Peak systolic (S'), early diastolic (E') medial and lateral mitral annular velocities were measured and medial and lateral E/E' ratio were calculated.[7] Full-volume multi-beat acquisitions from 4- and 2-chamber views were obtained for the assessment of 3D LV volumes and EF according to current guidelines using the cardiac 3 dimensional Quantification Advanced software (3DQ Advanced). TAPSE and S' velocity values were recorded as measures of RV function.[8] Speckle tracking was used to calculate global longitudinal strain using aCMQ software (*QLAB 10.0, iE33, Philips Healthcare, Best, The Netherlands*).[9] Sector size and depth were adjusted for each patient to achieve optimal visualiaation at the highest possible frame rate.

3. Cardiopulmonary Exercise Testing

Patients underwent CPET using maximal treadmill ergometry and dedicated ramp protocols (low, intermediate and high), under the direction of a specialist exercise physiologist (*Appendix Table 1-3)*. The exercise protocols were specifically designed for the study. The protocols were designed to accommodate patients with a range of exercise capacities with the overall aim of getting each patient to achieve a total exercise time of 8-12 minutes, as previously described.[10] The eventual protocol was chosen based on the estimated pre-test exercise tolerance. The total exercise time, peak oxygen consumption (VO2) and the percentage of predicted peak VO2 achieved were calculated and recorded.

The predicted peak VO2 was calculated using previously published equations.[11] A further adjustment is made based on the observed difference between peak VO2 during cycle as compared with treadmill ergometry. Studies have demonstrated that peak VO2 on cycling is approximately 90% of that observed during treadmill exercise.[11-13]

Appendix Table A. Low-intensity treadmill protocol detailing the stages of exercise.

Exercise	Time	Speed (mph)	Speed (kph)	Gradient
Stage 1	00:15	1	1.6	0
Stage 2	00:15	1.1	1.8	0.1
Stage 3	00:15	1.2	1.9	0.3
Stage 4	00:15	1.3	2.1	0.5
Stage 5	00:15	1.4	2.3	0.7
Stage 6	00:15	1.5	2.4	0.9
Stage 7	00:15	1.6	2.6	1
Stage 8	00:15	1.7	2.7	1.1
Stage 9	00:15	1.8	2.9	1.3
Stage 10	00:15	1.9	3.1	1.5
Stage 11	00:15	2	3.2	1.7
Stage 12	00:15	2.1	3.4	1.9
Stage 13	00:15	2.1	3.4	2
Stage 14	00:15	2.2	3.5	2.1
Stage 15	00:15	2.3	3.7	2.3
Stage 16	00:15	2.3	3.7	2.5
Stage 17	00:15	2.3	3.7	2.7
Stage 18	00:15	2.4	3.9	2.9
Stage 19	00:15	2.4	3.9	3
Stage 20	00:15	2.5	4.0	3.1
Stage 21	00:15	2.5	4.0	3.1
Stage 22	00:15	2.6	4.2	3.5
Stage 23	00:15	2.6	4.2	3.7
Stage 24	00:15	2.7	4.3	3.9
Stage 25	00:15	2.7	4.3	4
Stage 26	00:15	2.8	4.5	4.1
Stage 27	00:15	2.8	4.5	4.3
Stage 28	00:15	2.9	4.7	4.5
Stage 29	00:15	2.9	4.7	4.7
Stage 30	00:15	3	4.8	4.9
Stage 31	00:15	3	4.8	5
Stage 32	00:15	3.1	5.0	5.1
Stage 33	00:15	3.1	5.0	5.2
Stage 34	00:15	3.2	5.1	5.5
Stage 35	00:15	3.2	5.1	5.7
Stage 36	00:15	3.3	5.3	5.9
Stage 37	00:15	3.3	5.3	6
Stage 38	00:15	3.4	5.5	6.1
Stage 39	00:15	3.4	5.5	6.3
Stage 40	00:15	3.5	5.6	6.5
Stage 41	00:15	3.5	5.6	6.7
Stage 42	00:15	3.6	5.8	6.9
Stage 43	00:15	3.6	5.8	7
Stage 44	00:15	3.7	6.0	7.1
Stage 45	00:15	3.7	6.0	7.3
Stage 46	00:15	3.8	6.1	7.5

Appendix Table B. Intermediate-intensity treadmill protocol detailing the stages of exercise.

Exercise	Time	Speed (mph)	Speed (kph)	Gradient
Stage 1	00:15	1	1.6	0
Stage 2	00:15	1.1	1.8	0.2
Stage 3	00:15	1.2	1.9	0.5
Stage 4	00:15	1.3	2.1	0.7
Stage 5	00:15	1.4	2.3	1
Stage 6	00:15	1.5	2.4	1.2
Stage 7	00:15	1.5	2.4	1.5
Stage 8	00:15	1.6	2.6	1.7
Stage 9	00:15	1.7	2.7	2
Stage 10	00:15	1.8	2.9	2.1
Stage 11	00:15	1.9	3.1	2.3
Stage 12	00:15	2	3.2	2.5
Stage 13	00:15	2	3.2	2.7
Stage 14	00:15	2.1	3.4	3
Stage 15	00:15	2.2	3.5	3.1
Stage 16	00:15	2.3	3.7	3.3
Stage 17	00:15	2.4	3.9	3.5
Stage 18	00:15	2.5	4.0	3.7
Stage 19	00:15	2.5	4.0	3.9
Stage 20	00:15	2.6	4.2	4
Stage 21	00:15	2.7	4.3	4.1
Stage 22	00:15	2.8	4.5	4.3
Stage 23	00:15	2.9	4.7	4.5
Stage 24	00:15	3	4.8	4.7
Stage 25	00:15	3	4.8	4.9
Stage 26	00:15	3.1	5.0	5
Stage 27	00:15	3.2	5.1	5.1
Stage 28	00:15	3.3	5.3	5.3
Stage 29	00:15	3.4	5.5	5.5
Stage 30	00:15	3.5	5.6	5.7
Stage 31	00:15	3.5	5.6	5.9
Stage 32	00:15	3.6	5.8	6
Stage 33	00:15	3.7	6.0	6.3
Stage 34	00:15	3.8	6.1	6.7
Stage 35	00:15	3.9	6.3	7
Stage 36	00:15	4	6.4	7.3
Stage 37	00:15	4.1	6.6	7.7
Stage 38	00:15	4.2	6.8	8
Stage 39	00:15	4.3	6.9	8.3
Stage 40	00:15	4.4	7.1	8.7
Stage 41	00:15	4.5	7.2	9
Stage 42	00:15	4.6	7.4	9.3
Stage 43	00:15	4.7	7.6	9.7
Stage 44	00:15	4.8	7.7	10
Stage 45	00:15	4.9	7.9	10.3
Stage 46	00:15	5	8.0	10.7
Stage 47	00:15	5.1	8.2	11
Stage 48	00:15	5.2	8.4	11.3
Stage 49	00:15	5.3	8.5	11.7

Appendix Table C. High-intensity treadmill protocol detailing the stages of exercise.

Exercise	Time	Speed (mph)	Speed (kph)	Gradient
Stage 1	00:15	1	1.6	0
Stage 2	00:15	1.1	1.8	0.5
Stage 3	00:15	1.2	1.9	1
Stage 4	00:15	1.3	2.1	1.5
Stage 5	00:15	1.4	2.3	2
Stage 6	00:15	1.5	2.4	2.5
Stage 7	00:15	1.6	2.6	3
Stage 8	00:15	1.8	2.9	4
Stage 9	00:15	1.9	3.1	4.5
Stage 10	00:15	2	3.2	5
Stage 11	00:15	2.1	3.4	5.5
Stage 12	00:15	2.2	3.5	6
Stage 13	00:15	2.3	3.7	6.5
Stage 14	00:15	2.4	3.9	7
Stage 15	00:15	2.5	4.0	7.5
Stage 16	00:15	2.6	4.2	8
Stage 17	00:15	2.7	4.3	8.5
Stage 18	00:15	2.8	4.5	9
Stage 19	00:15	2.9	4.7	9.5
Stage 20	00:15	3	4.8	10
Stage 21	00:15	3.1	5.0	10.5
Stage 22	00:15	3.2	5.1	11
Stage 23	00:15	3.3	5.3	11.5
Stage 24	00:15	3.4	5.5	12
Stage 25	00:15	3.5	5.6	12.5
Stage 26	00:15	3.6	5.8	13
Stage 27	00:15	3.8	6.1	14
Stage 28	00:15	3.9	6.3	14.5
Stage 29	00:15	4	6.4	15
Stage 30	00:15	4.1	6.6	15.5
Stage 31	00:15	4.2	6.8	16
Stage 32	00:15	4.3	6.9	16.5
Stage 33	00:15	4.4	7.1	17
Stage 34	00:15	4.5	7.2	18
Stage 35	00:15	5	8.0	18
Stage 36	00:15	5.1	8.2	18.5
Stage 37	00:15	5.2	8.4	19
Stage 38	00:15	5.3	8.5	19.5
Stage 39	00:15	5.4	8.7	20
Stage 40	00:15	5.5	8.8	20.5
Stage 41	00:15	5.6	9.0	21
Stage 42	00:15	5.7	9.2	21.5
Stage 43	00:15	5.8	9.3	22
Stage 44	00:15	5.9	9.5	22.5
Stage 45	00:15	6	9.7	23
Stage 46	00:15	6.1	9.8	23.5
Stage 47	00:15	6.2	10.0	24
Stage 48	00:15	6.3	10.1	24.5
Stage 49	00:15	6.4	10.3	25

4. Symptom Assessment Questionnaire

Symptoms Score using any number between 0 and 9 With 0 meaning no problem or none, 5 meaning troublesome and 9 being severe	
**Breathlessness**	
Running (score 9 if you don't run because it makes you very breathless)	
On moderate exertion (eg:- walking quickly, climbing 2-3 flights of stairs)	
On mild exertion (eg:- walking slowly or doing light housework)	
On slight exertion (washing or dressing)	
Is walking impaired due to joint, nerve or muscle problems?	
Sitting at rest	
Do you get breathless lying flat?	
**Other Symptoms**	
Swelling of Ankles	
Swelling of Legs Above Ankles	
Are you troubled by tiredness during the day?	
Do you suffer much from anxiety?	
Do you feel depressed?	
Chest Pain on Exertion	
Dizziness	
Palpitations	
Muscle Aches & Pains	
Cough and/or Wheeze	
Other Symptom or Problem (Name)	
Other Symptom or Problem (Name)	
**Quality of Life (with 1 = very good, 5 = average, 9 = very bad)**	
How do you rate your health?	
How do you rate your overall quality of life?	
**Please also answer these questions "Yes" or "No" or Give a Number**	
How many pillows do you sleep with?	
Do you sometimes wake in the night fighting for breath?	
If so, how many nights in the last two months?	
Have you had any falls?	
If so, how many in the last two months?	
Have you had any blackouts?	
If so, how many in the last two months?	

TRED Trial. Version 1.0 25th November 2015

Appendix Figure 1. The symptom assessment questionnaire

5. Genetic Sequencing

Patients underwent targeted next-generation sequencing using the Illumina TruSight Cardio Sequencing kit, which includes 174 genes linked with inherited cardiac disease, on the NextSeq platform. Targeted deoxyribonucleic acid libraries were prepared and sequenced with paired-end reads of 150bp. Demultiplexing of sequence data was performed on NextSeq Control software or Bcl2FastQ conversion 2.16[14, 15] and resulting FastQ files subjected to quality control with the FastQC[16] v.0.10.14. Low quality reads (Q<20, window_size 5) were trimmed using PrinSeq[17] v0.20.4, and sequences aligned to the HG19 reference genome using BWA[18] v0.7.10. Picard[19] v1.115 and GATK[20] v3.2-2 were used to mark duplicate reads and perform local realignment around indels and base quality score recalibration. Bases covered by at least 10 reads with a mapping quality ≥10 and base quality ≥20 were denoted as "callable", i.e. adequately covered for variant calling with recommended GATK parameters. Variant calling was performed jointly with GATK HaplotypeCaller.

For all samples, variants were annotated using CardioClassifier.[21] ACMG class was determined using CardioClassifier[21] followed by manual curation of segregation, de novo and functional data from the literature and ClinVar. Truncating variants were defined as those resulting in nonsense, frameshift, or essential splice site mutations (VEP consequence: frameshift variant, stop gained, splice donor variant, splice acceptor variant) and non-truncating variants defined as those resulting in missense variants and inframe indels (VEP consequence: missense variant, inframe deletion, inframe insertion). Cases used in burden testing were matched by ethnicity: we selected those self-reporting as Caucasian and confirmed ethnicity by principal component analysis (PCA) analysed using PLINK v1.9 and HapMap3 as reference dataset.

Appendix Table D. Genes with an excess of rare variants in DCM

Gene symbol	Non-Truncating	Truncating	Transcript ID
BAG3	YES	YES	ENST00000369085
LMNA	YES	YES	ENST00000368300
TCAP	YES	YES	ENST00000309889
TNNC1	YES	YES	ENST00000232975
TNNT2	YES	YES	ENST00000367318
DSP	NO	YES	ENST00000379802
SCN5A	NO	YES	ENST00000333535
TTN	NO	YES	ENST00000589042
MYH7	YES	NO	ENST00000355349
RBM20	YES	YES	ENST00000369519
VCL	NO	YES	ENST00000211998
TPM1	YES	NO	ENST00000403994

The genes with an excess of rare variants in DCM cohorts as compared to ExAC reference samples,[22] as well as BAG3 which has also been demonstrated to be an important gene associated with DCM.[23]

Appendix Table E. List of likely pathogenic or pathogenic rare variants amongst patients enrolled

Gene	HGVSc	Consequence	ExAC_freq	ACMG_class	ACMG rules
TTN	c.79523G>A	Stop gained	0	Likely pathogenic	PVS1_strong,PM2
TTN	c.85090C>T	Stop gained	0	Likely pathogenic	PVS1_strong,PM2
TTN	c.14245C>T	Stop gained	0	Likely pathogenic	PVS1_strong,PM2
TTN	c.81262_81269delCAGATGCT	Frameshift variant	0	Likely pathogenic	PVS1_strong,PM2
TTN	c.89846_89847delCA	Frameshift variant	0	Likely pathogenic	PVS1_strong,PM2
TTN	c.85470delA	Frameshift variant	0	Likely pathogenic	PVS1_strong,PM2
TTN	c.10303+2T>C	Splice donor variant	8.25E-06	Likely pathogenic	PVS1_strong,PM2
TTN	c.86821+2T>A	Splice donor variant	8.91E-06	Likely pathogenic	PVS1_strong,PM2, PP1
TTN	c.55037delG	Frameshift variant	0	Likely pathogenic	PVS1_strong,PM2
TTN	c.97062delA	Frameshift variant	0	Likely pathogenic	PVS1_strong,PM2
TTN	c.12643_12644delCA	Frameshift variant	0	Likely pathogenic	PVS1_strong,PM2

*ACMG class was determined using CardioClassifier[21] followed by manual curation of segregation, de novo and functional data from the literature and ClinVar.

Appendix Table F. List of additional rare variants classified as of uncertain significance amongst patients enrolled

Gene	HGVSc	Consequence	ExAC_freq	ACMG_class	ACMG rules
MYH7	c.3676C>G	Misense variant	0	VUS	PM2
TNNT2	c.415C>T	Misense variant	0	VUS	PM2,PP3
LMNA	c.1750C>T	Misense variant	9.30E-06	VUS	PM2,PP3

*ACMG class was determined using CardioClassifier[21] followed by manual curation of segregation, de novo and functional data from the literature and ClinVar.

6. Treatment withdrawal algorithm

The treatment withdrawal algorithm was constructed by the investigator team which included senior heart failure clinicians and clinical trialists. As part of the planning process, the opinions of independent experts within the field and outside of the investigator group were sought and the protocol underwent external, peer review at the time of the funding application. The overall aim was to mimic what might happen in clinical practice if the clinician and patient agreed to a trial of therapy withdrawal.

Loop diuretics were withdrawn first as the identification of those prone to congestion was considered a priority to minimise the risk to which such patients were exposed. Mineralocorticoid receptor antagonists (MRAs) were withdrawn next. The position of MRAs in guidelines,[24, 25] beneath ACE inhibitors and beta-blockers, and the recommendation to use them in the sub-group patients who remain symptomatic from heart failure with a LVEF<35% were important in determining this decision.

ACE inhibitors and beta-blockers were considered to have similar priority in the withdrawal algorithm. The mechanisms responsible for remodelling are likely to play variable roles amongst different patients. Adrenergic stimulation, targeted by beta-blockers, appears to be one important pathway. The renin-angiotensin-aldosterone axis is another important pathway, playing a central role in the development of myocyte hypertrophy and interstitial fibrosis. On the balance of the information available, it was decided to perform phased withdrawal of beta-blockers before ACE inhibitors. The mechanisms that underpin remodelling and relapse are important areas for future research.

Down-titration of medications was performed in a phased process over weeks and months. This enabled the investigators to identify signs of deterioration in cardiac function after a reduction in dose and removed the possible risks of complete medication withdrawal in these circumstances.

7. Kaplan-Meier curve of the cross-over cohort

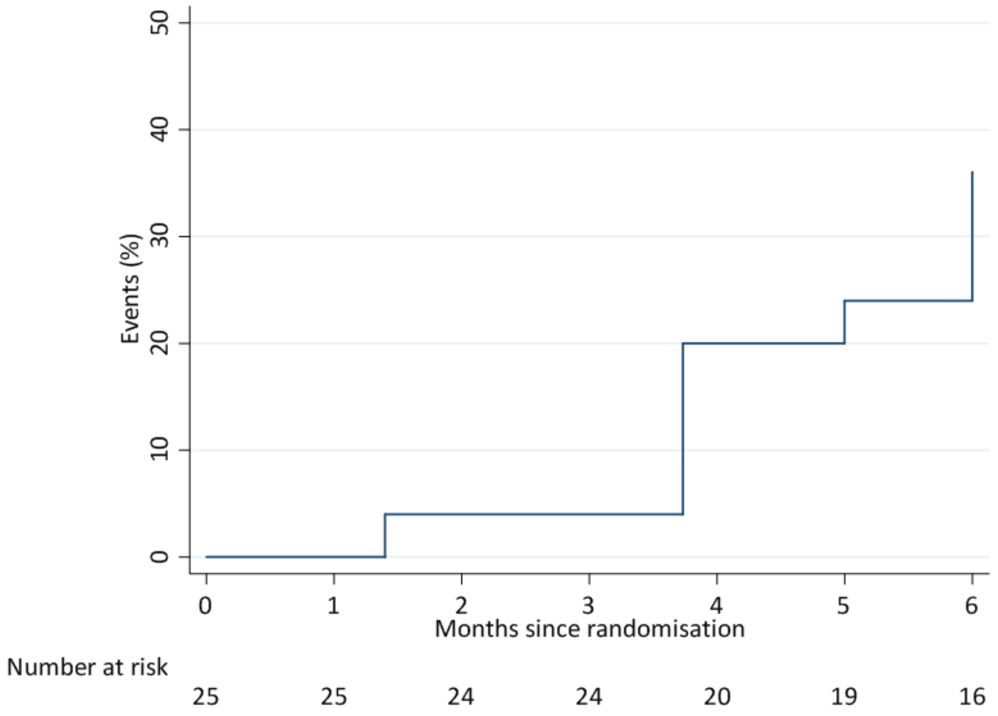

Appendix Figure 2. Kaplan Meier plot of time to the primary outcome after crossover to therapy withdrawal following 6 months in the control group

8. Primary end-point data

Appendix Table G. Change in end-point components between baseline and follow-up for those patients who met criteria for primary end-point

End-point number	Baseline			End-point			Comments
	LVEF (%)	LVEDVi (ml/m^2)	NT-pro-BNP (ng/L)	LVEF (%)	LVEDVi (ml/m^2)	NT-pro-BNP (ng/L)	
1	53	77	42	40	83	38	
2	68	65	78	76	76	441	Developed peripheral oedema
3	72	65	76	48	100	324	
4	66	87	126	54	106	202	
5	52	62	44	40	83	29	
6	62	84	160	57	82	1393	Onset of AF
7	63	88	96	47	95	78	
8	64	82	159	54	94	177	
9	51	82	128	51	97	509	
10	62	74	82	28	92	411	
11	61	65	125	48	81	208	
12	57	75	53	48	83	519	
13	57	75	135	46	83	1417	
14	55	91	240	35	107	960	
15	52	91	29	41	102	18	
16*	51	49	162	42	62	501	Developed shortness of breath (NYHA II)
17	58	92	33	52	102	133	
18	54	97	161	43	99	51	
19	57	98	85	60	113	50	NT-pro-BNP subsequently increased to 172ng/L
20	66	96	201	32	96	1734	Onset of AF with shortness of breath (NYHA II)

*Baseline and follow-up studies performed using 3-dimensional echocardiography.

9. Standard deviation of the continuous variables including the proportional hazard models in Table 2

Continuous variables from Table 2	Standard Deviation
Time since initial diagnosis, years	3.5
LVEF at initial diagnosis, %	9.0
Heart rate, bpm	11.0
Systolic blood pressureP, mmHg	11.3
Diastolic blood pressure, mmHg	9.7
QRS duration, ms	21.1
Log NT-proBNP, ng/L	0.74
LVEF at enrolment, %	6.0
LVEDVi at enrolment, ml/m^2	15.0
LAVi at enrolment, ml/m^2	9.3
Global radial strain	0.10
Global circumferential strain	0.028
Global longitudinal strain	0.023
Native T1, ms	35.7
ECV, %	0.032
Peak VO2, ml/kg/min	6.8
% predicted peak VO2, %	17.6

10. Characteristics of patients based on the occurrence of relapse

Appendix Table H. Characteristics of patients who underwent therapy withdrawal divided by the occurrence of the primary end-point

	Relapse (n=20)	No Relapse (n=30)	P
**Demographics**			
Median Age (IQR), yrs	62 (52,66)	49 (44,62)	0.0245
Men, n (%)	11 (55)	22 (73)	0.2293
**Previous cardiovascular history**			
Time since initial DCM diagnosis, months	64 (33,103)	56 (29,95	0.6205
LVEF at initial diagnosis, %	22 (18,33)	29 (20,34)	0.1558
Absolute improvement in LVEF, %	33 (28,40)	30 (25,39)	0.4634
Time since LVEF>50%, months	29 (14, 51)	24 (11, 49)	0.8352
Previous heart failure admission, n (%)	11 (55)	20 (67)	0.5532
Previous moderate alcohol excess, n (%)	8 (40)	8 (27)	0.3662
Previous atrial fibrillation, n (%)	3 (15)	8 (27)	0.4895
Previous hypertension, n (%)	1 (5)	3 (10)	0.6411
Diabetes mellitus, n (%)	1 (5)	0 (0)	0.4000
**Aetiology**			
Idiopathic	13 (65)	21 (70)	1.000
Familial	3 (15)	4 (13)	
Environmental insult	4 (20)	5 (17)	
TTNtv	4 (20)	7 (23)	1.000
**Medications**			
ACE inhibitor/ARB, n (%)	20 (100)	30 (100)	N/A
Beta-Blocker, n (%)	19 (95)	25 (83)	0.3811
Mineralocorticoid receptor antagonist, n (%)	15 (75)	9 (30)	0.0034
Loop Diuretic, n (%)	4 (20)	2 (7)	0.2017
**Clinical characteristics at therapy withdrawal**			
Heart rate, beats per minute	70 (61,75)	64 (59,74)	0.2007
Systolic blood pressure, mmHg	123 (117,132)	125 (118,132)	0.7362
Diastolic blood pressure, mmHg	72 (67,78)	72 (66,80)	0.8351
Left bundle branch block, n (%)	3 (15)	4 (13)	1.0000
QRS duration, ms	95 (88,105)	97 (86,108)	0.8974
NT-pro-BNP, ng/l	111 (65,160)	51 (32,97	0.0189
**CMR variables at therapy withdrawal**			
LVEDVi, ml/m^2	83 (66,90)	83 (66,90)	0.8122
LVEF, %	58 (54,64)	60 (55,66)	0.3162
LV mass index, g/m^2	61 (52,74)	71 (62,77)	0.1323
RVEDVi, ml/m^2 *	78 (69,84)	79 (64,92)	0.6471
RVEF, % *	59 (53,63)	58 (54,53)	0.7675
LAVi, ml/m^2	41 (36,45)	40 (31,48)	0.9526
Late gadolinium enhancement, presence *	8 (42)	12 (41)	1.000
Native T1 time, ms *	1288 (1272,1313)	1293 (1266,1318)	0.7678
Extracellular volume, % *	26 (25,28)	25 (23,28)	0.2095
Global radial strain#	0.23 (0.18,0.29)	0.31 (0.23,0.40)	0.0069
Global circumferential strain#	-0.15 (-0.16,-0.13)	-0.15 (-0.18,-0.13)	0.3650
Global longitudinal strain*	-0.14 (-0.15,-0.11)	-0.14 (-0.15,-.0.12)	0.4312
**CPET at therapy withdrawal**			
Peak VO_2, ml/kg/min ^	23 (20,30)	29 (23,34)	0.0523
Percentage of predicted peak VO_2, % ^	98 (78,107)	91 (81,103)	0.7615
Exercise time, seconds ^	563 (519,631)	591 (517,635)	0.6853
**Symptom Questionnaires Scores**			
Kansas City Cardiomyopathy Questionnaire	95 (94,99)	96 (93,100)	0.6005
Symptom assessment questionnaire	9 (4,13)	11 (6,17)	0.1626

Data presented as median (IQR) or n (%). Mann-Whitney test used to compare continuous data and Fisher's exact test used to compare categorical data. Characteristics are taken immediately before the start of therapy withdrawal.

Missing data: *n=2 did not undergo CMR at baseline due to contraindications; #n=3; in addition to 2 pts who did not undergo CMR, global circumferential and radial strain unable to be calculated from images available for 1 pt; ^ n=4 did not undergo CPET at baseline due to musculoskeletal pain or injury

DCM: dilated cardiomyopathy; LAVi: left atrial volume indexed to body surface area (BSA); LV: left ventricular; LVEDVi: left ventricular end diastolic volume indexed to BSA; LVEF: left ventricular ejection fraction; RVEDVi: right ventricular end diastolic volume indexed to BSA; *TTNtv:* truncating variant in *TTN;* VO_2: oxygen consumption

11. Characteristics of patients who demonstrated no evidence of deterioration

Appendix Table I. Characteristics of patients divided by the presence or absence of deterioration after starting therapy withdrawal.

	Deterioration or restart meds (n=34)	No deterioration (n=16)	P
**Demographics**			
Median Age (IQR), yrs	59 (47, 64)	48 (43,56)	0.0582
Men, n (%)	21 (62)	12 (75)	0.5241
**Previous cardiovascular history**			
Time since initial DCM diagnosis, months	63 (40,107)	44 (25,71)	0.1832
LVEF at initial diagnosis, %	25 (19,33)	31 (23,34)	0.1926
Absolute improvement in LVEF, %	33 (26,40)	28 (23,35)	0.1287
Time since LVEF>50%, months	29 (12,49)	21 (11,31)	0.2612
Previous heart failure admission, n (%)	18 (53)	13 (81)	0.0678
Previous moderate alcohol excess, n (%)	11 (32)	5 (31)	1.0000
Previous atrial fibrillation, n (%)	5 (15)	6 (38)	0.1399
Previous hypertension, n (%)	3 (9)	1 (6)	1.0000
Diabetes mellitus, n (%)	1 (3)	0 (0)	1.0000
Smoker, n (%)	2 (6)	1 (6)	1.0000
**Aetiology**			
Idiopathic, n (%)	23 (68)	11 (69)	1.0000
Familial, n (%)	5 (15)	2 (13)	
Environmental insult, n (%)	6 (18)	3 (19)	
TTNtv, n (%)	8 (24)	3 (19)	1.0000
**Medications**			
ACE inhibitor /ARB, n (%)	34 (100)	16 (100)	N/A
Beta-blocker, n (%)	32 (94)	12 (75)	0.0741
Mineralocorticoid receptor antagonist, n (%)	21 (62)	3 (19)	0.0063
Loop diuretic, n (%)	5 (15)	1 (6)	0.6498
**Clinical characteristics at therapy withdrawal**			
Heart rate, beats per minute	65 (60,75)	64 (59,72)	0.3275
Systolic blood pressure, mmHg	123 (117,131)	125 (121,134)	0.2745
Diastolic blood pressure, mmHg	71 (68,77)	74 (63,85)	0.6544
Left bundle branch block, n (%)	5 (15)	2 (13)	1.0000
QRS duration, ms	95 (86,104)	99 (88,115)	0.4045
NT-pro-BNP, ng/l	97 (50,136)	44 (31,65)	0.0080
**CMR variables at therapy withdrawal**			
LVEDVi, ml/m2	84 (65,93)	83 (67,87)	0.3493
LVEF, %	61 (55,65)	59 (55,65)	0.7625
LV mass index, g/m2	65 (53,74)	74 (63,78)	0.1901
RVEDVi, ml/m2 *	77 (64,85)	85 (67,92)	0.3737
RVEF, % *	58 (55,64)	57 (53,61)	0.2376
LAVi, ml/m2	41 (34,46)	40 (38,46)	0.9007
Late gadolinium enhancement, presence *	14 (41)	6 (38)	1.0000
Native T1 time, ms *	1291 (1273,1313)	1289 (1253,1321)	0.8153
Extracellular volume, % *	26 (24,28)	25 (21,28)	0.3734
Global radial strain#	0.25 (0.21,0.31)	0.34 (0.28,0.44)	0.0145
Global circumferential strain#	-0.15 (-0.17 -0.13)	-0.15 (-0.18,-0.14)	0.4123
Global longitudinal strain*	-0.14 (-0.15,-0.12)	-0.13 (-0.15,-0.12)	0.8752
**CPET at therapy withdrawal**			
Peak VO2, ml/kg/min ^	27 (22,32)	28 (22,30)	0.6563
Percentage of predicted peak VO2, % ^	98 (81,108)	86 (78,95)	0.1639
Exercise time, seconds ^	584 (530,639)	585 (513,628)	0.8697
**Symptom questionnaires scores**			
Kansas City Cardiomyopathy Questionnaire	96 (94,100)	95 (90,99)	0.9242
Symptom assessment questionnaire	9 (4,14)	12 (10,16)	0.1901

Patients classified as ‘No deterioration’ are those who began therapy withdrawal remained asymptomatic, off medication and either finished the study with a left ventricular ejection fraction within the normal range or, had a ≤3% absolute reduction in left ventricular ejection fraction (the limit of expected interstudy variability[1]) or an increase in left ventricular ejection fraction.

Data presented as median (IQR) or n (%). Mann-Whitney test used to compare continuous data and Fisher's exact test used to compare categorical data. Characteristics are taken immediately before the start of therapy withdrawal.

Missing data: *n=2 did not undergo CMR at baseline due to contraindications; [#]n=3; in addition to 2 pts who did not undergo CMR, global circumferential and radial strain unable to be calculated from images available for 1 pt; ^ n=4 did not undergo CPET at baseline due to musculoskeletal pain or injury

DCM: dilated cardiomyopathy; LAVi: left atrial volume indexed to body surface area (BSA); LV: left ventricular; LVEDVi: left ventricular end diastolic volume indexed to BSA; LVEF: left ventricular ejection fraction; RVEDVi: right ventricular end diastolic volume indexed to BSA; *TTNtv:* truncating variant in *TTN;* VO_2: oxygen consumption

Appendix References

1. Budge LP, Helms AS, Salerno M, Kramer CM, Epstein FH, Bilchick KC. MR cine DENSE dyssynchrony parameters for the evaluation of heart failure: comparison with myocardial tissue tagging. *JACC Cardiovasc Imaging* 2012; **5**: 789-97.
2. Aletras AH, Ding S, Balaban RS, Wen H. DENSE: displacement encoding with stimulated echoes in cardiac functional MRI. *J Magn Reson* 1999; **137**: 247-52.
3. Gulati A, Ismail TF, Jabbour A, et al. Clinical utility and prognostic value of left atrial volume assessment by cardiovascular magnetic resonance in non-ischaemic dilated cardiomyopathy. *Eur J Heart Fail* 2013; **15**: 660-70.
4. Flett AS, Hayward MP, Ashworth MT, et al. Equilibrium contrast cardiovascular magnetic resonance for the measurement of diffuse myocardial fibrosis: preliminary validation in humans. *Circulation* 2010; **122**: 138-44.
5. Suever JD, Wehner GJ, Haggerty CM, et al. Simplified post processing of cine DENSE cardiovascular magnetic resonance for quantification of cardiac mechanics. *J Cardiovasc Magn Reson* 2014; **16**: 94.
6. Lang RM, Bierig M, Devereux RB, et al. Recommendations for chamber quantification. *J Am Soc Echocardiogr* 2005; **18**: 1440-63.
7. Lang RM, Badano LP, Mor-Avi V, et al. Recommendations for cardiac chamber quantification by echocardiography in adults. *Eur Heart J Cardiovasc Imaging* 2015; **16**: 233-70.
8. Rudski LG, Lai WW, Afilalo J, et al. Guidelines for the echocardiographic assessment of the right heart in adults. *J Am Soc Echocardiogr* 2010; **23**: 685-713; quiz 86-8.
9. Mor-Avi V, Lang RM, Badano LP, et al. Current and evolving echocardiographic techniques for the quantitative evaluation of cardiac mechanics. *Eur J Echocardiogr* 2011; **12**: 167-205.
10. Myers J, Do D, Herbert W, Ribisl P, Froelicher VF. A nomogram to predict exercise capacity from a specific activity questionnaire and clinical data. *Am J Cardiol* 1994; **73**: 591-6.
11. Wasserman K. Principles of exercise testing and interpretation : including pathophysiology and clinical applications. 5th ed. Philadelphia: Wolters Kluwer Health/Lippincott Williams & Wilkins; 2012.
12. Hermansen L, Saltin B. Oxygen uptake during maximal treadmill and bicycle exercise. *J Appl Physiol* 1969; **26**: 31-7.
13. Faulkner JA, Roberts DE, Elk RL, Conway J. Cardiovascular responses to submaximum and maximum effort cycling and running. *J Appl Physiol* 1971; **30**: 457-61.
14. Zannad F, Alla F, Dousset B, Perez A, Pitt B. Limitation of excessive extracellular matrix turnover may contribute to survival benefit of spironolactone therapy in patients with congestive heart failure: insights from the randomized aldactone evaluation study (RALES). Rales Investigators. *Circulation* 2000; **102**: 2700-6.
15. Klappacher G, Franzen P, Haab D, et al. Measuring extracellular matrix turnover in the serum of patients with idiopathic or ischemic dilated cardiomyopathy and impact on diagnosis and prognosis. *Am J Cardiol* 1995; **75**: 913-8.
16. Haugaa KH, Goebel B, Dahlslett T, et al. Risk assessment of ventricular arrhythmias in patients with nonischemic dilated cardiomyopathy by strain echocardiography. *J Am Soc Echocardiogr* 2012; **25**: 667-73.
17. Schmieder R, Edwards R. Quality control and preprocessing of metagenomic datasets. *Bioinformatics* 2011; **27**: 863-4.
18. Li H, Durbin R. Fast and accurate long-read alignment with Burrows-Wheeler transform. *Bioinformatics* 2010; **26**: 589-95.
19. Kioka H, Yamada T, Mine T, et al. Prediction of sudden death in patients with mild-to-moderate chronic heart failure by using cardiac iodine-123 metaiodobenzylguanidine imaging. *Heart* 2007; **93**: 1213-8.
20. McKenna A, Hanna M, Banks E, et al. The Genome Analysis Toolkit: a MapReduce framework for analyzing next-generation DNA sequencing data. *Genome Res* 2010; **20**: 1297-303.
21. Whiffin N, Walsh R, Govind R, et al. CardioClassifier: disease- and gene-specific computational decision support for clinical genome interpretation. *Genet Med* 2018.
22. Walsh R, Thomson KL, Ware JS, et al. Reassessment of Mendelian gene pathogenicity using 7,855 cardiomyopathy cases and 60,706 reference samples. *Genet Med* 2017; **19**: 192-203.
23. Villard E, Perret C, Gary F, et al. A genome-wide association study identifies two loci associated with heart failure due to dilated cardiomyopathy. *Eur Heart J* 2011; **32**: 1065-76.
24. Yancy CW, Jessup M, Bozkurt B, et al. 2013 ACCF/AHA guideline for the management of heart failure. *Circulation* 2013; **128**: e240-327.
25. Ponikowski P, Voors AA, Anker SD, et al. 2016 ESC Guidelines for the diagnosis and treatment of acute and chronic heart failure. *Eur Heart J* 2016; **37**: 2129-200.
